# Small incision lenticule extraction (SMILE) history, fundamentals of a new refractive surgery technique and clinical outcomes

**DOI:** 10.1186/s40662-014-0003-1

**Published:** 2014-10-16

**Authors:** Dan Z Reinstein, Timothy J Archer, Marine Gobbe

**Affiliations:** London Vision Clinic, 138 Harley Street, London, W1G 7LA UK; Department of Ophthalmology, Columbia University Medical Center, New York, NY USA; Centre Hospitalier National d’Ophtalmologie, Paris, France

**Keywords:** Small incision lenticule extraction (SMILE), Laser in situ keratomileusis (LASIK), Lenticule, Cap, Dry eye, Corneal sensation, Corneal innervation, Corneal biomechanics, Endokeratophakia

## Abstract

This review summarizes the current status of the small incision lenticule extraction (SMILE) procedure. Following the early work by Sekundo et al. and Shah et al., SMILE has become increasingly popular. The accuracy of the creation of the lenticule with the VisuMax femtosecond laser (Carl Zeiss Meditec) has been verified using very high-frequency (VHF) digital ultrasound and optical coherence tomography (OCT). Visual and refractive outcomes have been shown to be similar to those achieved with laser in situ keratomileusis (LASIK), notably in a large population reported by Hjortdal, Vestergaard et al. Safety in terms of the change in corrected distance visual acuity (CDVA) has also been shown to be similar to LASIK. It was expected that there would be less postoperative dry eye after SMILE compared to LASIK because the anterior stroma is disturbed only by the small incision, meaning that the anterior corneal nerves should be less affected. A number of studies have demonstrated a lower reduction and faster recovery of corneal sensation after SMILE than LASIK. Some studies have also used confocal microscopy to demonstrate a lower decrease in subbasal nerve fiber density after SMILE than LASIK. The potential biomechanical advantages of SMILE have been modeled by Reinstein et al. based on the non-linearity of tensile strength through the stroma. Studies have reported a similar change in Ocular Response Analyzer (Reichert) parameters after SMILE and LASIK, however, these have previously been shown to be unreliable as a representation of corneal biomechanics. Retreatment options after SMILE are discussed. Tissue addition applications of the SMILE procedure are also discussed including the potential for cryo-preservation of the lenticule for later reimplantation (Mohamed-Noriega, Angunawela, Lim et al.), and a new procedure referred to as endokeratophakia in which a myopic SMILE lenticule is implanted into a hyperopic patient (Pradhan et al.). Finally, studies reporting microdistortions in Bowman’s layer and corneal wound healing responses are also described.

## Additional non-English abstract (French, Francais)

Cette revue résume les connaissances actuelles sur la procedure SMILE (small incision lenticule extraction). Apres les travaux préliminaires des groupes de Sekundo et al. et de Shah et al., la procedure SMILE est désormais devenue courante. Le laser femtosecond VisuMax (Carl Zeiss Meditec) permet une découpe précise du lenticule, comme cela a été vérifié par ultrasound numérique à tres haute fréquence (Artemis) et par OCT. Il a été démontré, entre autres par Hjortdal, Vestergaard, et al. dans leur etude sur une population large, que les résultats visuels et refractifs sont comparables à ceux du LASIK, et que le risque de perte de lignes d’acuité corrigée est aussi semblable à celui du LASIK. Avec SMILE, contrairement au LASIK, la partie antérieure du stroma étant seulement découpée par une incision de petite taille, les nerfs cornéens antérieurs sont moins touchés et par consequent, le taux d’yeux secs en phase post-opératoire devrait etre plus faible après SMILE qu’apres LASIK. Quelques études ont effectivement démontré que la sensation cornéenne était moins réduite après SMILE qu’après LASIK et que la récupération de la sensation cornéenne était plus rapide. Quelques études ont aussi utilisé la microscopie confocale pour prouver qu’il y avait une diminution plus faible de la densité des fibres nerveuses de la couche sub-basale après SMILE qu’apres LASIK. Au niveau biomécanique, Reinstein et al. ont proposé un modèle basé sur la non-linéarité de la force de tension du stroma, mettant en avant les avantages potentiels de SMILE. Certaines études ont comparé les paramètres mesurés par l’Ocular Response Analayzer (Reichert) et rapporté un changement similaire après SMILE ou LASIK; cependant, on sait que ces paramètres ne peuvent pas etre pris en compte pour représenter la biomécanique cornéenne. Les options de re-traitement après SMILE sont présentées, ainsi que la possibilité d’ajouter du tisuu cornéen: par example, le potential de cryo-preservation du lenticule pour une ré-implantation ultérieure (Mohamed-Noriega, Angunawela, Lim et al.), et une nouvelle procedure nommée endokératophakie dans laquelle un lenticule SMILE est implanté dans la cornée d’un oeil hypermétrope (Pradhan et al.). Pour finir, des études rapportant des micro-distortions dans la couche de Bowmans et des études sur le processus de reparation de la cornée sont décrites.

## Introduction

Ever since femtosecond lasers were first introduced into refractive surgery, the ultimate goal has been to create an intrastromal lenticule that can then be manually removed as a single piece thereby circumventing the need for incremental photoablation by an excimer laser. A precursor to modern refractive lenticule extraction (ReLEx) was first described in 1996 using a picosecond laser to generate an intrastromal lenticule that was removed manually after lifting the flap [1,2], however, significant manual dissection was required leading to an irregular surface. The switch to femtosecond improved the precision [3] and studies were performed in rabbit eyes in 1998 [4] and in partially sighted eyes in 2003 [5], but these initial studies were not followed up with further clinical trials.

Following the introduction of the VisuMax femtosecond laser (Carl Zeiss Meditec, Jena, Germany) in 2007 [6], the intrastromal lenticule method was reintroduced in a procedure called Femtosecond Lenticule Extraction (FLEx). The 6-month results of the first 10 fully seeing eyes treated were published in 2008 [7] and results of a larger population have since been reported [8,9]. The refractive results were similar to those observed in laser in situ keratomileusis (LASIK), but visual recovery time was longer due to the lack of optimization in energy parameters and scan modes; further refinements have led to much improved visual recovery times [10].

**Figure 1:**
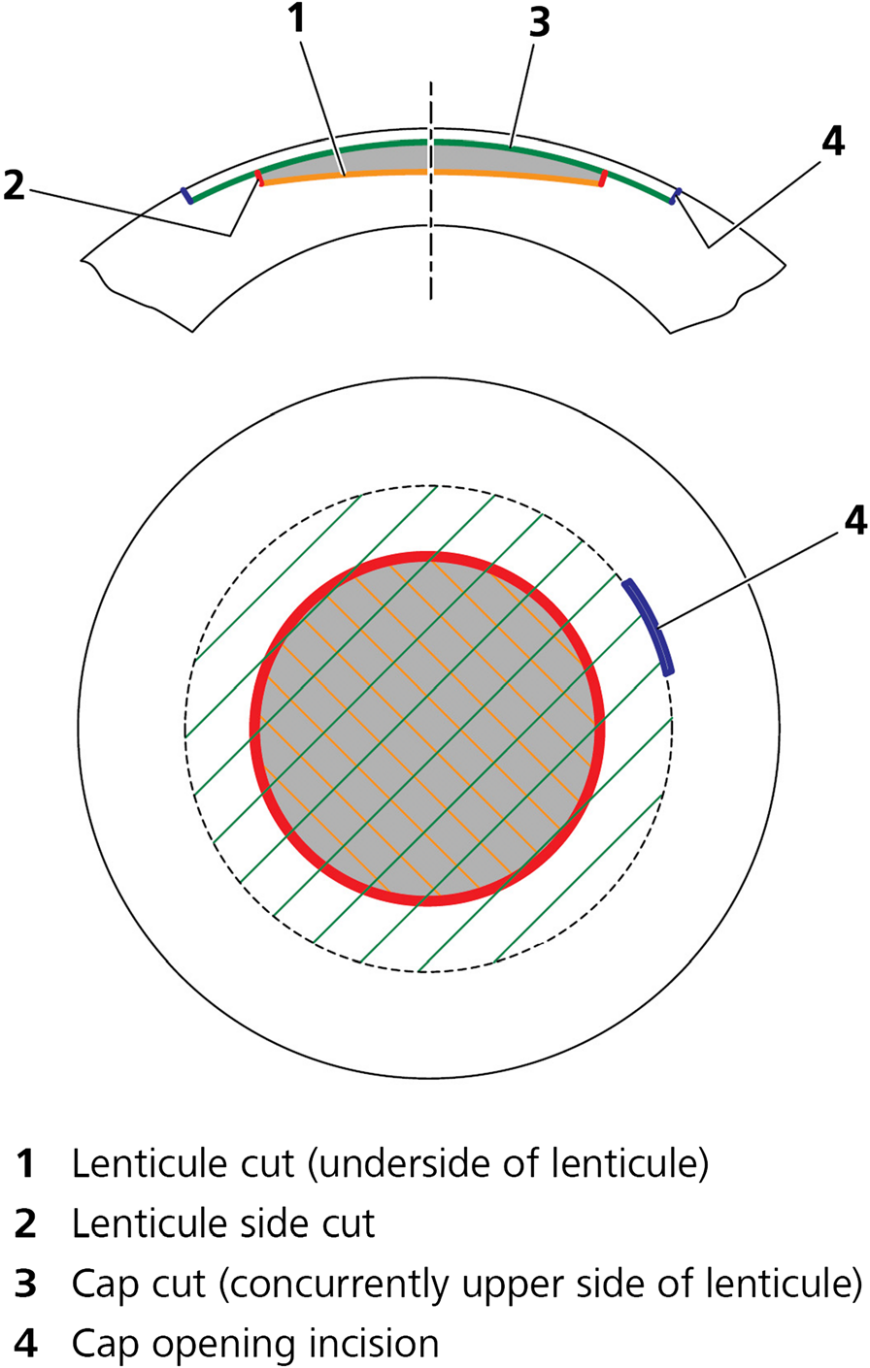
**Incision geometry of the SMILE procedure.** The lenticule cut (1) is performed (the underside of the lenticule), followed by the lenticule sidecuts (2). Next, the cap interface (3) is created (the upper side of the lenticule), and finally a 2–3 mm small incision (4) is created supero-temporally. The lenticule interfaces are dissected using a flap separator and the lenticule is extracted manually, all via the small incision.

Following the successful implementation of FLEx, a new procedure called Small Incision Lenticule Extraction (SMILE) was developed. This procedure involves passing a dissector through a small 2–3 mm incision to separate the lenticular interfaces and allow the lenticule to be removed, as shown in Figure 1, thus eliminating the need to create a flap. The SMILE procedure is now gaining popularity following the results of the first prospective trials [11–13] and a growing number of other related studies are now being published, which are discussed below (this article is focused on SMILE, and so further references for FLEx are not included).

## Review

### A new surgical approach - SMILE

During the SMILE procedure, the patient is raised to the contact glass of the femtosecond laser. At the moment of contact between the individually calibrated curved contact glass and the cornea, a meniscus tear film appears, at which point the patient is able to see the fixation target very clearly because the vergence of the fixation beam is focused according to the patient’s refraction. At this point, the surgeon instructs the patient to look directly at the green light and once in position, the corneal suction ports are activated to fixate the eye in this position. In this way, the patient essentially auto-centrates the visual axis and hence the corneal vertex to the vertex of the contact glass which is centered to the laser system and the center of the lenticule to be created [14]. The centration is confirmed by the surgeon by comparing the relative positions of the corneal reflex and pupil center to the placido eye image obtained by the Atlas topography scan (Carl Zeiss Meditec, Jena, Germany). If however, the surgeon is not satisfied with the centration of the docking, the suction is released and the docking procedure is repeated.

Due to the corneal suction and curved contact glass the intraocular pressure (IOP) is raised to only 70–80 mmHg [15,16], low enough that intraocular circulation and the patient’s vision is maintained throughout the procedure. The lower interface of the intrastromal lenticule is created first (using an out-to-in direction to maximize the time without blurring the patient’s central vision), followed by the upper interface of the lenticule (using an in-to-out direction), known as the cap, and finally a 2–3 mm tunnel incision (usually supero-temporal) that links the cap interface to the corneal surface (Figure 1). Total suction time is approximately 35 seconds and is independent of refractive error treated as there are always the same cuts, but simply further apart for higher corrections.

The patient is then moved to the surgical microscope for the lenticule separation and extraction part of the procedure. Surgical technique varies to a degree between surgeons, but the following describes the main elements. The small incision is opened and the upper and lower edges of the lenticule are delineated, so that the tissue planes are well defined. The upper interface is usually separated first using a standard lamellar corneal surgical technique of waving the instrument back and forth (although it does not matter if the interfaces are separated in the reverse order). A number of different interface separation instruments have been developed, which seem to be converging on a design with a blunt circular tip. The lower layer is then dissected in a similar fashion. During separation, some surgeons prefer to hold the eye steady to have better control over the force being used when separating the surgical planes. Once both layers have been separated, the lenticule is removed from the cornea using a pair of retinal micro-forceps or can be extracted directly from within the pocket with the latest versions of the lenticule separating instrument.

At the end of the procedure, any redundant portions of the cap need to be distributed evenly to the periphery using a dry micro-spear to avoid mud-crack type microfolds in the cap, which results from the length mismatch between bed and cap after lenticule extraction. This can be done either at the built-in slit-lamp of the VisuMax or the patient can be taken to a standard slit-lamp. Our preference is to sit the patient at a slit-lamp so that fluorescein dye imaging can be performed using a bright slit-lamp with cobalt blue illumination to better appreciate any tension lines that may be present in redundant areas of the cap.

When planning the treatment, the following parameters can be selected by the surgeon: cap thickness, cap diameter, cap sidecut angle, refractive correction, lenticule diameter (optical zone), lenticule sidecut angle, and the minimum lenticule thickness (so that the lower lenticule interface can be easily differentiated from the upper interface).

### Refractive outcomes of SMILE and precision of lenticule creation

There are now a number of studies [17–22] reporting the visual and refractive outcomes after SMILE, which have demonstrated that these are similar to those achieved as set out in Table 1. The disadvantage of SMILE currently is its slightly slower visual recovery compared to LASIK, where its day one visual acuity is lower than LASIK [17], although significant improvements have been made in this area by using different energy and spot spacing settings [10].

**Table 1:** **Visual and refractive outcomes after SMILE**

**Study**	**Eyes**	**Follow-up**	**Preop SEQ (D)**	**Postop SEQ (D)**	**±0.50 D**	**20/20 CDVA preop**	**UDVA 20/20 or better postop**	**UDVA 20/25 or better postop**	**Loss 2 lines CDVA**
Sekundo 2011 [11]	91	6 months	−4.75 ± 1.56	−0.01 ± 0.49	80%	---	84%	92%	1.1%
Shah 2011 [12]	51	6 months	−4.87 ± 2.16	+0.03 ± 0.30	91%	67%	62%	79%	0.0%
			−1.75 to −10.00	−0.75 to +0.75					
Vestergaard 2012 [17]	127	3 months	−7.18 ± 1.57	−0.09 ± 0.45	77%	---	37%	73%	0.4%
			−1.63 to −11.50	−1.63 to +1.38					
Hjortdal 2012 [13]	670	3 months	−7.19 ± 1.30	−0.25 ± 0.44	80%	88%	61%	84%	2.4%
			−1.63 to −9.88	−2.13 to +1.38					
Wang 2013 [18]	88	3 months	---	−0.11 ± 0.29	---	---	100%	---	0.0%
Kamiya 2014 [19]	26	6 months	−4.21 ± 1.63	+0.01	100%	100%	96%	---	0.0%
			−1.25 to −8.25						
Sekundo 2014 [20]	54	1 year	−4.68 ± 1.29	−0.19 ± 0.19	92%	98%	88%	98%	0.0%
			−2.00 to −9.00	−1.00 to +0.50					
Agca 2014 [21]	40	1 year	−4.03 ± 1.61	−0.33 ± 0.25	95%	---	65%	95%	0.0%
Lin 2014 [22]	60	3 months	−5.13 ± 1.75	−0.09 ± 0.38	---	---	85%	93%	1.7%
			−1.75 to −7.75	−1.25 to +0.75					

SMILE: small incision lenticule extraction; Preop: preoperative; Postop: postoperative; SEQ: spherical equivalent refraction; CDVA: corrected distance visual acuity; UDVA: uncorrected distance visual acuity.

The safety of SMILE has also been shown to be very good with a very low percentage of eyes with a loss of 2 or more lines corrected distance visual acuity (CDVA). A large population analysis of 1,800 eyes by Ivarsen et al. [23] reported the incidence of complications including epithelial abrasions (6%), small tears at the incision (1.8%), and difficult lenticule extraction (1.9%). The cap was perforated in 4 eyes (0.22%), and a major tear occurred in 1 eye (0.06%); however, none of these patients had late visual symptoms. In 0.8% (14 eyes), suction was lost during surgery. Postoperative complications included trace haze (8%), epithelial dryness on day 1 (5%), interface inflammation secondary to central abrasion (0.3%), and minor interface infiltrates (0.3%); these complications had an impact on CDVA at 3 months in only 1 case. Irregular corneal topography occurred in 1.0% of eyes (18 eyes), resulting in reduced 3-month CDVA (12 eyes) or ghost images (6 eyes). Topography-guided custom ablation [24] was effective in improving cases of irregular astigmatism. Another complication unique to SMILE that has been reported is a lenticule remnant being left in the interface causing irregular astigmatism [25]. This type of complication has been successfully treated using trans-epithelial phototherapeutic keratectomy (PTK) [26].

In terms of the induction of higher order aberrations, Sekundo et al. [20], using a 5-mm diameter analysis zone, have reported an increase in total higher order root mean square (RMS) of 0.10 μm and an increase in spherical aberration of 0.05 μm (OSA notation). Two studies have compared the induction of higher order aberrations between SMILE and LASIK [21,22]. Agca et al. [21], using a 6-mm diameter analysis zone, found the induction of total higher order RMS (increase by 0.14 μm) and spherical aberration (increase by 0.07 μm, OSA notation) to be similar between SMILE and LASIK. Lin et al. [22] reported an increase in total higher order RMS of 0.12 μm and an increase in spherical aberration of 0.27 μm (although analysis zone and notation for aberrations were not reported) 3 months after SMILE. These changes in higher order aberrations were found to be less than for the LASIK population for which the total higher order RMS increased by 0.21 μm and spherical aberration increased by 0.69 μm.

The overall efficacy of SMILE demonstrated above is dependent on the precision with which the lenticule can be created by the femtosecond laser, so it is important to investigate whether the intended lenticule dimensions are achieved. Currently, SMILE has only been performed using the VisuMax femtosecond laser for which the flap thickness reproducibility has been reported to be 3.8 μm [27], 5.0 μm [28], 5.1 μm [29], 7.9 μm [6,30], 13.9 μm [31], and in the range of 7.5-14.4 μm (for different flap thicknesses) [32]. There are now also studies reporting a similar outcome for cap thickness in SMILE (equivalent to flap thickness in LASIK) ranging between 4.4-9.0 μm (see Table 2) [33–36]. These studies also demonstrated very good accuracy of the cap thickness with mean accuracy between −1.2 and +5.0 μm (see Table 2) [28,33–36].

**Table 2:** **Accuracy and reproducibility of SMILE cap thickness**

**Author**	**Measurement method**	**Eyes**	**Accuracy (** **μm)**	**Reproducibility (** **μm)**
Reinstein 2013 [33]	Artemis VHF digital ultrasound	70	−0.7	4.4
Zhao 2013 [34]	RTVue AS-OCT	54	−1.2	5.1
Vestergaard 2014 [35]	Heidelberg Spectralis AS-OCT	34	+4	9
Ozgurhan 2013 [28]	Visante AS-OCT	66	+4.6	5.2
Tay 2012 [36]	RTVue AS-OCT	95	+5.0	---

SMILE: small incision lenticule extraction; VHF: very high-frequency; AS-OCT: anterior segment optical coherence tomography.

The variation between studies can largely be explained by the different instruments used to obtain the cap thickness measurements. The Artemis very high-frequency (VHF) digital ultrasound scanner (ArcScan Inc, Morrison, Colo) has a flap thickness repeatability of 1.68 μm [37], compared with 4.2-7.4 μm with the RTVue optical coherence tomography (OCT) (Optovue, Fremont, CA) [38,39], and 4.8-8.7 μm with the Visante OCT (Carl Zeiss Meditec, Jena, Germany) [40–42]. The reason for the difference in repeatability between VHF digital ultrasound and OCT is that the flap/cap interface is identified manually by clicking on the OCT image, whereas this interface is measured directly from the peak of the ultrasound scan data. For example, one study showed the 95% confidence interval for inter-observer measurement error with the RTVue OCT to be ±20 μm [36]. The positive bias found in three of the four OCT studies can also be explained by epithelial thickening, which is known to occur after myopic tissue removal and has been described after SMILE [35,43].

Another advantage of VHF digital ultrasound is that it can generate a 10 mm map of flap/cap thickness so that uniformity can be evaluated. In our study we found a slight vertical asymmetry with the cap 2.3 μm thinner than intended 2 mm superiorly and 6.5 μm thicker than intended 2 mm inferiorly [33]. The cap thickness was found to be highly uniform compared to microkeratome flaps: for example, the within-eye variation of 4.3 μm for the VisuMax was 60% better than the 10.7 μm for the standard Hansatome and 10.4 μm for the zero compression Hansatome [44].

We have also used VHF digital ultrasound to measure the accuracy of the thickness of the SMILE lenticule [43]. The readout central lenticule depth was 8.2 μm thicker on average than the Artemis measured stromal thickness change. If this error were due to VisuMax cutting accuracy, there would have to be an error in only one of the interfaces (if the same error occurred in both interfaces, then there would not be an error in lenticule thickness). However, as described above, the cap thickness was accurate with a central accuracy of −0.7 μm [33]. Therefore, if the lenticule thickness difference was due to the VisuMax cutting accuracy, the error must have been in the lower interface of the lenticule. However, the accuracy in our previous study was found to be similar for cap thicknesses between 80 and 140 μm [33]. This provides evidence that the accuracy of the VisuMax does not vary with depth (although this needs to be confirmed for depths at which the lower interface of the lenticule is created). Hence, the lower interface would have similar accuracy as that measured for the upper interface, and therefore the lenticule thickness error would not appear to be due to cutting accuracy.

This difference can be partially explained by alignment errors between the pre- and post-operative scans. As the corneal pachymetry is thinnest centrally and radially thicker toward the periphery, any misalignment in the postop scan will mean that the thinnest point of the postop scan will not be aligned with the thinnest point of the preop scan. This means that in the majority of cases, an alignment error will tend to underestimate the change in stromal thickness, as was observed in this population.

However, it is unlikely that these alignment errors could explain a systematic difference of 8 μm because the pachymetric progression of the central stroma is relatively gradual [45]. Therefore, this study seems to provide evidence for some central stromal expansion caused by biomechanical changes occurring after SMILE. One possible mechanism could be that the lamellae severed by the lenticule in between the residual bed and the cap might be recoiling and causing expansion of the stroma as they are no longer under tension, similar to the known peripheral stromal expansion after LASIK [46,47]. This expansion might be keeping the bottom lamellae of the cap slightly apart from the top lamellae of the residual bed. It seems unlikely that there would be any reason for the stroma in the residual bed or the cap to be expanding as they are still under tension. For example, the high accuracy of cap thickness as described above provides evidence for biomechanical stability within the cap. Therefore, it would appear that the cut lamellae within the interface are causing a small separation between the stroma above and below the interface.

### Ocular surface and tear film condition after SMILE

The cornea is one of the most densely innervated peripheral tissues in humans. Nerve bundles within the anterior stroma grow radially inward from the periphery towards the central cornea [48,49]. The nerves then penetrate Bowman’s layer and create a dense network of nerve fibers, known as the subbasal nerve plexus, by branching both vertically and horizontally between Bowman’s layer and basal epithelial cells. In LASIK, subbasal nerve bundles and superficial stromal nerve bundles in the flap interface are cut by the microkeratome or femtosecond laser, with only nerves entering the flap through the hinge region being spared. Subsequent excimer laser ablation severs further stromal nerve fiber bundles. Therefore, corneal sensation is decreased while the nerves regenerate. The lower corneal sensation can lead to a reduction in the blink rate, resulting in epitheliopathy (known as LASIK-induced neurotrophic epitheliopathy) due to the increased ocular surface exposure and patients feel ‘dry eye’ [50,51]. While there are also other contributing factors, it is generally accepted that corneal denervation is the largest factor [52,53].

Therefore, following the introduction of SMILE, there was an expectation that SMILE may demonstrate an improvement in postoperative dry eye compared to LASIK given that the anterior cornea is left untouched other than the small incision. A number of studies have investigated this by measuring corneal sensation [54–61] using aesthesiometry and corneal innervation using confocal microscopy [57,61,62].

In our study including 156 eyes, corneal sensation was reduced in the early postoperative period after SMILE, but recovered to baseline in 76% of eyes by 3 months and in 89% of eyes by 6 months [54]. In this study, we also performed a literature review of studies reporting the corneal sensation after LASIK and plotted our results against the average of the LASIK studies. Our SMILE results compared favorably to LASIK with less reduction in central corneal sensation at all time points, particularly in the first 3 months.

Similar results have been reported in other SMILE studies. Wei et al. [55] found significantly higher central corneal sensation in the SMILE group (n = 61) compared with the LASIK group (n = 54) at 1 week, 1 month and 3 months. Central corneal sensation decreased only slightly at 1 week and recovered to baseline 3 months after SMILE, whereas it had not reached baseline in the LASIK group. Similar results were found in a larger study by the same group [56].

Vestergaard et al. [57] performed a contra-lateral eye study comparing central corneal sensation after FLEx and SMILE in 35 myopic patients. At the 6-month time point, mean central corneal sensation was found to have returned to the baseline level in the SMILE group (1.0 mm less than baseline, p > 0.05). In contrast, mean central corneal sensation was 3.8 mm less than baseline in the FLEx group (p < 0.05) and was statistically significantly lower than the SMILE group.

Demirok et al. [58] performed a contra-lateral eye study comparing central corneal sensation after LASIK and SMILE in 28 myopic patients over a 6 months follow-up period. Mean central corneal sensation was reduced after both SMILE and LASIK at 1 week, 1 month, and 3 months, however it was statistically significantly higher in the SMILE group at each of these time points. Central corneal sensation had returned to baseline levels at the 6-month time point in both groups. Although there was a difference in corneal sensation, other dry eye parameters were not affected including tear break-up time, Schirmer test, and tear film osmolarity.

Li et al. [59,60] compared the change in central corneal sensation between SMILE (n = 38) and LASIK (n = 31) over a 6 months follow-up period. Mean central corneal sensation was reduced after both SMILE and LASIK at 1 week, 1 month, 3 months, and 6 months, however it was statistically significantly higher in the SMILE group at each of these time points. As with the previous study, although there was a difference in corneal sensation, there were no real differences between groups for other dry eye parameters, such as tear break-up time, Schirmer test, and the Ocular Surface Disease Index (OSDI) questionnaire. Similar results were found by the same group in a second study [61].

**Figure 2:**
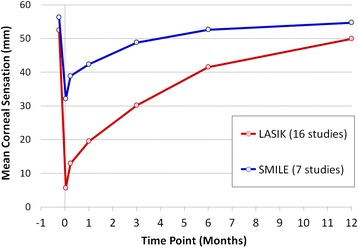
**Line graph showing the mean central corneal sensation over the 12-month follow-up period averaged across 7 SMILE studies and 16 LASIK studies following a review of the peer-reviewed literature.**

Figure 2 shows the average corneal sensation (across all seven studies after SMILE) plotted over time [54–61]. For comparison, the graph also shows the average corneal sensation (across sixteen studies [54] after LASIK where the Cochet-Bonnet aesthesiometer had been used) plotted over time.

A few studies have also investigated the change in corneal innervation using confocal microscopy. Vestergaard et al. [57] demonstrated that the decrease in corneal nerves was greater after LASIK compared with SMILE at 6 months. Li et al. [61] found that the decrease in subbasal nerve fiber density was less severe in the first 3 months after SMILE than after LASIK. Similarly, Mohamed-Noriega et al. found less nerve damage and faster nerve recovery in rabbit eyes 4 weeks after SMILE compared to LASIK [62].

Finally, a recent study by Xu et al. [63] compared dry eye parameters between SMILE and LASIK. They found that all parameters became worse in the early postoperative period in both groups, however, Schirmer’s test, tear break up time, and the McMonnies score were all better in the SMILE group.

It would not be expected for SMILE to completely eliminate dry eye symptoms after surgery as there appear to be other mechanisms that also contribute to dry eye after laser refractive surgery [52,53]. The other factor that explains some of the variation in results is the cap thickness that was used in the different studies; thinner cap thicknesses (100–110 μm) will mean that the lenticule is created more anteriorly and so would be expected to have a greater impact on the corneal nerve plexus than using thicker caps.

### Potential biomechanical advantages of SMILE

Another potential benefit of the SMILE procedure is increased biomechanical stability due to the absence of a flap. Firstly, it is known that vertical cuts (e.g. flap sidecut) have more biomechanical impact than horizontal cuts. Recently, Knox Cartwright et al. [64] performed a study on human cadaver eyes that compared the corneal strain produced by a LASIK flap, a sidecut only, and a delamination cut only, with each incision type performed at both 90 μm and 160 μm. Table 3 summarizes the results, which found that the increase in strain was equivalent between a LASIK flap and a sidecut alone at both depths with a significantly greater increase for the 160 μm depth. In contrast, the increase in strain after a delamination cut only was lower than after a LASIK flap or sidecut only. Also, the strain did not increase when a delamination cut only was performed at the greater 160 μm depth. A similar result has also been found in a study by Medeiros et al. [65], who showed in pig eyes that there were significantly greater biomechanical changes following the creation of a thick flap of 300 μm compared to a thin flap of 100 μm.

**Table 3:** **Comparison of the increase in corneal strain induced by a LASIK flap, sidecut only, and delamination only**

	**90** **μm**	**160** **μm**
LASIK Flap	9%	32%
Sidecut Only	9%	33%
Delamination Only	5%	5%

Percentage increase in central corneal strain (to an intraocular pressure change from 15 mmHg to 15.5 mmHg) after the creation of a LASIK flap, a sidecut or delamination at both 90 μm and 160 μm. (Data obtained from [64]).

Applying this finding to SMILE, since no anterior corneal sidecut is created, there will be less of an increase in corneal strain in SMILE compared to thin flap LASIK and a significant difference in corneal strain compared to LASIK with a thicker flap.

**Figure 3:**
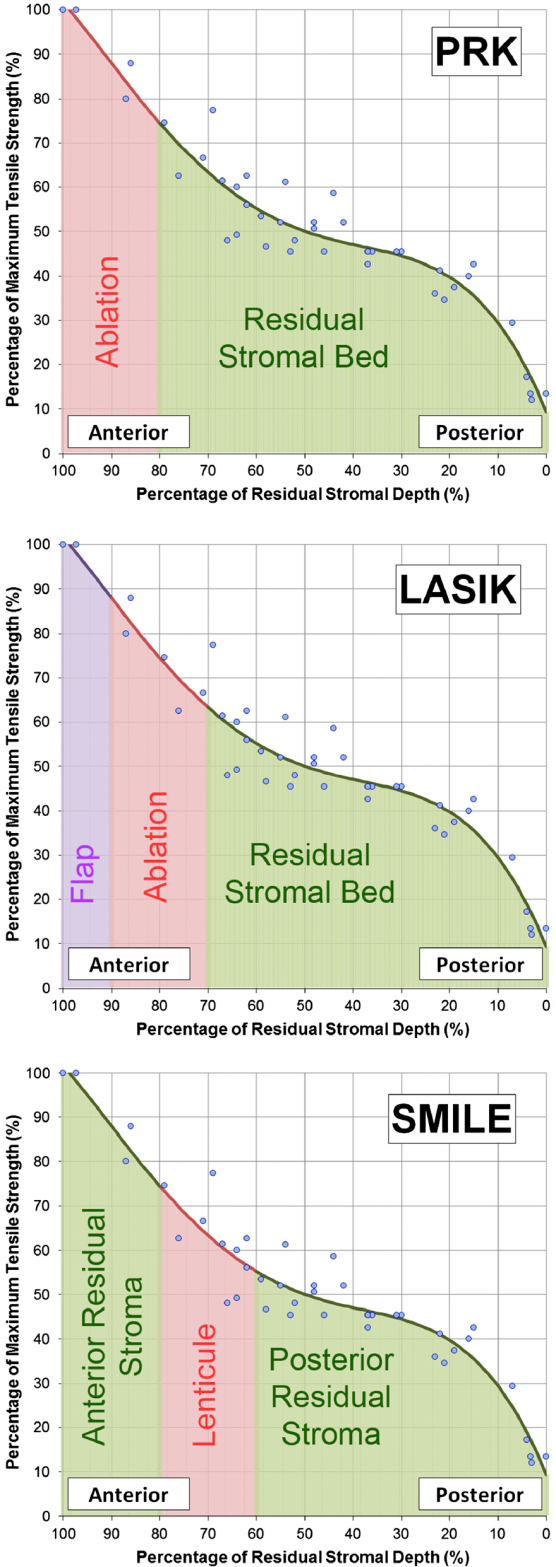
**Scatter plot of the percentage of maximum cohesive tensile strength against the percentage of residual stromal depth using data from the study by Randleman et al. [** 66 **].** A fourth order polynomial regression line was fit to the data and this equation was integrated to calculate the area under the curve for the relevant stromal depths after photorefractive keratectomy (PRK), laser in-situ keratomileusis (LASIK), and small incision lenticule extraction (SMILE) as demonstrated by the green shaded regions. The red areas represent the tissue removed (excimer laser ablation/lenticule extraction) and the purple area in LASIK represents the LASIK flap. Reprinted with permission from [71].

The second biomechanical difference is due to the fact that anterior stromal lamellae are stronger than posterior stromal lamellae. Randleman et al. [66] demonstrated that the cohesive tensile strength (i.e., how strongly the stromal lamellae are held together) of the stroma decreases from anterior to posterior within the central corneal region (Figure 3). In an experiment in which the cohesive tensile strength was measured for strips of stromal lamellae cut from different depths within donor corneoscleral buttons, a strong negative correlation was found between stromal depth and cohesive tensile strength. The anterior 40% of the central corneal stroma was found to be the strongest region of the cornea, whereas the posterior 60% of the stroma was at least 50% weaker.

In addition to cohesive tensile strength, tangential tensile strength (i.e., stiffness along the stromal lamellae) and shear strength (i.e., resistance to torsional forces) have both been found to vary with depth in the stroma. Kohlhaas et al. [67] and Scarcelli et al. [68] found that the tangential tensile strength was greater for the anterior stroma than posterior stroma, each using different methodology. Petsche et al. [69] found a similar result for transverse shear strength to decrease with stromal depth. The same group have used nonlinear optical high-resolution macroscopy to image the three-dimensional distribution of transverse collagen fibers and have shown that the non-linearity of tensile strength through the stroma is caused by the greater interconnectivity of the collagen fibers in the anterior stroma compared to the posterior stroma where the collagen fibers lie in parallel to each other [70].

Applying this knowledge to SMILE, since the anterior stroma remains uncut, the strongest part of the stroma continues to contribute to the strength of the cornea postoperatively, in contrast to both photorefractive keratectomy (PRK) and LASIK where the strongest anterior stroma is affected. We recently developed a mathematical model based directly on the Randleman [66] depth-dependent tensile strength data to calculate the postoperative tensile strength and compared this between PRK, LASIK and SMILE [71]. We now suggest that this total tensile strength value should replace residual stromal thickness as the limiting factor for corneal refractive surgery.

In the model, we performed non-linear regression analysis on the Randleman [66] data and calculated the total tensile strength of the cornea as the area under the regression line by integration. The total tensile strength after PRK, LASIK and SMILE was then calculated as the area under the regression line for the depths of the stroma that remain uncut in each type of procedure (see Figure 3). The model demonstrated that the postoperative tensile strength would be greater after SMILE than after both PRK and LASIK. For example, for a central corneal thickness of 550 μm, the postoperative relative total tensile strength reached 60% for an ablation depth of 73 μm in LASIK (flap thickness of 110 μm, approximately −5.75 D), 132 μm in PRK (approximately −10.00 D), and 175 μm in SMILE (cap thickness of 130 μm, approximately −13.50 D), translating to a 7.75 D difference between LASIK and SMILE for a cornea of the same postoperative relative total tensile strength.

In summary, considering the safety of subtractive corneal refractive surgical procedures in terms of tensile strength represents a paradigm shift away from classical residual stromal thickness limits. The residual thickness based safety of corneal laser refractive surgery should be thought of at least in terms of total residual uncut stroma. Ideally, a parameter such as total tensile strength, which takes the nonlinearity of the strength of the stroma into account, seems more appropriate.

Measuring the biomechanical differences between SMILE and LASIK in vivo is a difficult challenge as currently there are very few instruments designed for this purpose. There are four studies where the Ocular Response Analyzer (Reichert Inc, Depew, NY) has been used to generate corneal hysteresis (CH) and corneal resistance factor (CRF) and all showed that CH and CRF were reduced after SMILE [35,72–74]. In three contra-lateral eye studies there was no difference in either CH or CRF between the SMILE and LASIK groups [35,72,73], while one study found that CH and CRF were slightly greater after SMILE than LASIK (p < 0.02) [74]. These results do not agree with the expected increased biomechanical strength after SMILE as described above. However, it is likely that CH and CRF are not ideal parameters for measuring corneal biomechanics [75] given that many studies show no change in CH and CRF after cross-linking [76]. It is also well-known that CH and CRF are correlated with corneal pachymetry [77], so it would be expected for CH and CRF to be reduced after SMILE due to tissue removal.

### Retreatments after SMILE

There are a number of different options for performing retreatments after SMILE, with the choice often dictated by the cap thickness that was used for the primary procedure. If a thin cap thickness (100–110 μm) had been used, then a femtosecond laser can be used to create a sidecut only to convert the cap into a flap, although this limits the optical zone that can be used. Alternatively, there are other options available in the VisuMax software referred to as Circle to convert the cap into a flap with a larger diameter than the original cap. These have been described and the ease of lifting the flap with the different options has been investigated [78].

If the cap thickness was thicker, then a thin flap LASIK procedure can be performed. The limiting factor for this option is whether a new LASIK interface can be safely created (a) without crossing the existing cap interface and potentially creating slivers that are difficult to handle, and (b) avoiding the creation of a cryptic buttonhole (also known as gas breakthrough) by the interface crossing into the epithelium (particularly as the epithelium will have thickened after the primary SMILE procedure). Ideally, a direct measurement of the existing cap interface and epithelial thickness would be performed before the retreatment.

Otherwise, a PRK procedure can be performed. In the future, it may be possible to perform another SMILE procedure either above or below the existing interface. Another possible future alternative is intra-stromal arcuate keratotomy incisions, which may be a good option for small astigmatic corrections.

### Tissue addition applications

The fact that the SMILE lenticule is extracted as a single piece opens up the possibility of using the lenticule for other purposes. It has been suggested that refractive lenticules might be stored so that re-implantation can be performed at a later date if needed [79,80]. This was proposed as a method for restoring tissue in ectatic corneas, or to provide an opportunity for reversing the myopic correction in a patient progressing to presbyopia [81]. Re-implantation of the refractive lenticule (under a flap) has been demonstrated in rabbits having been cryopreserved for one month [80].

Alternatively, there is also the potential for performing the keyhole intrastromal form of keratophakia first described by Jose Ignacio Barraquer in 1980 [82] in which a disc of donor corneal tissue is lathed to the appropriate refractive power and inserted into a manually created intrastromal pocket. This was Barraquer’s idea for a minimally invasive form of his keratophakia procedure whereby the donor refractive lens is inserted under a cap created by a microkeratome [83], or epikeratophakia where the donor refractive lens is sutured onto the de-epithelialized cornea and then the epithelium is allowed to cover the lenticule [84].

The SMILE procedure can therefore be used to create the donor lenticule of Barraquer’s pocket intrastromal keratophakia procedure, utilizing a refractive lenticule from one patient and re-implanting it intrastromally into a different patient through a small incision. This was demonstrated in a rabbit by Liu et al. [85]. The first endokeratophakia procedure in a human was described by Pradhan et al. [86] where a −10.00 D lenticule was removed from a myopic patient, set aside in McCarey-Kaufman (MK) medium storage, and inserted into a patient with +11.25 D of hyperopia and sensory exotropia. After 6 months, the spherical equivalent refraction had been reduced by +5.25 D and the cornea was clear. The reason for only achieving 50% correction was found to be that a significant proportion of the curvature change afforded by the implanted lenticule manifested on the posterior surface, meaning that the majority of the effect intended by this curvature change was lost given the similar refractive index between the stroma and the aqueous humor in the anterior chamber.

### Other biological and corneal optical observations in SMILE

One study has described microdistortions in Bowman’s layer after SMILE [87] identified by OCT, but with no clinically significant corneal striae at the slit-lamp. However, these microdistortions did not have an impact on visual acuity or quality. Central microdistortions can be minimized by distending the cap immediately at the end of the procedure as described earlier.

Another study investigated corneal wound healing and inflammatory responses in rabbits after SMILE and compared to LASIK [88]. In this study, SMILE induced less keratocyte apoptosis, proliferation and inflammation compared with femtosecond laser LASIK. This therefore suggests that SMILE may be associated with a slightly lower degree of regression than LASIK.

Another difference found between SMILE and LASIK is the light intensity of the corneal backscatter in the anterior stroma using in vivo confocal microscopy [89]. This study found the backscattered light intensity to be higher for SMILE than LASIK in the first 3 months after surgery due to the extracellular matrix and activated keratocytes and this was linked to the slower visual recovery observed after SMILE. The authors postulated possible causes as the greater femtosecond energy delivered to the cornea in SMILE, the fact that two femtosecond lamellar cut surfaces come face to face (as opposed to one surface being sculpted by an excimer laser), and the increased surgical maneuvers required in SMILE.

## Conclusions

The evolution of SMILE, a flapless intrastromal keyhole keratomileusis procedure, has introduced a new method for corneal refractive surgery. The visual and refractive outcomes of the procedure have been shown to be similar to LASIK, while there is increasing evidence for the benefits of SMILE over LASIK by leaving the anterior stroma intact including superior biomechanics and faster recovery of dry eye and corneal nerve reinnervation.

## Abbreviations

ReLEx: Refractive lenticule extraction
FLEx: Femtosecond lenticule extraction
SMILE: Small incision lenticule extraction
LASIK: Laser in situ keratomileusis
PRK: Photorefractive keratectomy
PTK: Phototherapeutic keratectomy
IOP: Intraocular pressure
CDVA: Corrected distance visual acuity
UDVA: Uncorrected distance visual acuity
VHF: Very high-frequency
OCT: Optical coherence tomography
SEQ: Spherical equivalent refraction
CH: Corneal hysteresis
CRF: Corneal resistance factor

## References

[1] Krueger RR, Juhasz T, Gualano A, Marchi V (1998). The picosecond laser for nonmechanical laser in situ keratomileusis. J Refract Surg.

[2] Ito M, Quantock AJ, Malhan S, Schanzlin DJ, Krueger RR (1996). Picosecond laser in situ keratomileusis with a 1053-nm Nd:YLF laser. J Refract Surg.

[3] Kurtz RM, Horvath C, Liu HH, Krueger RR, Juhasz T (1998). Lamellar refractive surgery with scanned intrastromal picosecond and femtosecond laser pulses in animal eyes. J Refract Surg.

[4] Heisterkamp A, Mamom T, Kermani O, Drommer W, Welling H, Ertmer W, Lubatschowski H (2003). Intrastromal refractive surgery with ultrashort laser pulses: in vivo study on the rabbit eye. Graefes Arch Clin Exp Ophthalmol.

[5] Ratkay-Traub I, Ferincz IE, Juhasz T, Kurtz RM, Krueger RR (2003). First clinical results with the femtosecond neodynium-glass laser in refractive surgery. J Refract Surg.

[6] Reinstein DZ, Archer TJ, Gobbe M, Johnson N (2010). Accuracy and reproducibility of Artemis central flap thickness and visual outcomes of LASIK with the Carl zeiss meditec VisuMax femtosecond laser and MEL 80 excimer laser platforms. J Refract Surg.

[7] Sekundo W, Kunert K, Russmann C, Gille A, Bissmann W, Stobrawa G, Sticker M, Bischoff M, Blum M (2008). First efficacy and safety study of femtosecond lenticule extraction for the correction of myopia: six-month results. J Cataract Refract Surg.

[8] Blum M, Kunert KS, Engelbrecht C, Dawczynski J, Sekundo W (2010). Femtosecond lenticule extraction (FLEx) - Results after 12 months in myopic astigmatism. Klin Monbl Augenheilkd.

[9] Vestergaard A, Ivarsen A, Asp S, Hjortdal JØ (2013). Femtosecond (FS) laser vision correction procedure for moderate to high myopia: a prospective study of ReLEx(®) flex and comparison with a retrospective study of FS-laser in situ keratomileusis. Acta Ophthalmol.

[10] Shah R, Shah S (2011). Effect of scanning patterns on the results of femtosecond laser lenticule extraction refractive surgery. J Cataract Refract Surg.

[11] Sekundo W, Kunert KS, Blum M (2011). Small incision corneal refractive surgery using the small incision lenticule extraction (SMILE) procedure for the correction of myopia and myopic astigmatism: results of a 6 month prospective study. Br J Ophthalmol.

[12] Shah R, Shah S, Sengupta S (2011). Results of small incision lenticule extraction: All-in-one femtosecond laser refractive surgery. J Cataract Refract Surg.

[13] Hjortdal JO, Vestergaard AH, Ivarsen A, Ragunathan S, Asp S (2012). Predictors for the outcome of small-incision lenticule extraction for Myopia. J Refract Surg.

[14] Pande M, Hillman JS (1993). Optical zone centration in keratorefractive surgery. Entrance pupil center, visual axis, coaxially sighted corneal reflex, or geometric corneal center?. Ophthalmology.

[15] Vetter JM, Holzer MP, Teping C, Weingartner WE, Gericke A, Stoffelns B, Pfeiffer N, Sekundo W (2011). Intraocular pressure during corneal flap preparation: comparison among four femtosecond lasers in porcine eyes. J Refract Surg.

[16] Vetter JM, Faust M, Gericke A, Pfeiffer N, Weingartner WE, Sekundo W (2012). Intraocular pressure measurements during flap preparation using 2 femtosecond lasers and 1 microkeratome in human donor eyes. J Cataract Refract Surg.

[17] Vestergaard A, Ivarsen AR, Asp S, Hjortdal JO (2012). Small-incision lenticule extraction for moderate to high myopia: predictability, safety, and patient satisfaction. J Cataract Refract Surg.

[18] Wang Y, Bao XL, Tang X, Zuo T, Geng WL, Jin Y (2013). Clinical study of femtosecond laser corneal small incision lenticule extraction for correction of myopia and myopic astigmatism. Zhonghua Yan Ke Za Zhi.

[19] Kamiya K, Shimizu K, Igarashi A, Kobashi H (2014). Visual and refractive outcomes of femtosecond lenticule extraction and small-incision lenticule extraction for myopia. Am J Ophthalmol.

[20] Sekundo W, Gertnere J, Bertelmann T, Solomatin I (2014). One-year refractive results, contrast sensitivity, high-order aberrations and complications after myopic small-incision lenticule extraction (ReLEx SMILE). Graefes Arch Clin Exp Ophthalmol.

[21] Agca A, Demirok A, Cankaya KI, Yasa D, Demircan A, Yildirim Y, Ozkaya A, Yilmaz OF (2014). Comparison of visual acuity and higher-order aberrations after femtosecond lenticule extraction and small-incision lenticule extraction. Cont Lens Anterior Eye.

[22] Lin F, Xu Y, Yang Y (2014). Comparison of the visual results after SMILE and femtosecond laser-assisted LASIK for myopia. J Refract Surg.

[23] Ivarsen A, Asp S, Hjortdal J (2014). Safety and complications of more than 1500 small-incision lenticule extraction procedures. Ophthalmology.

[24] Reinstein DZ, Archer TJ, Gobbe M (2009). Combined corneal topography and corneal wavefront data in the treatment of corneal irregularity and refractive error in LASIK or PRK using the Carl Zeiss Meditec MEL80 and CRS Master. J Refract Surg.

[25] Dong Z, Zhou X (2013). Irregular astigmatism after femtosecond laser refractive lenticule extraction. J Cataract Refract Surg.

[26] Reinstein DZ, Archer TJ, Dickeson ZI, Gobbe M (2014). Trans-epithelial phototherapeutic keratectomy protocol for treating irregular astigmatism based population on epithelial thickness measurements by Artemis very high-frequency digital ultrasound. J Refract Surg.

[27] Ju WK, Lee JH, Chung TY, Chung ES (2011). Reproducibility of LASIK flap thickness using the zeiss femtosecond laser measured postoperatively by optical coherence tomography. J Refract Surg.

[28] Ozgurhan EB, Agca A, Bozkurt E, Gencer B, Celik U, Cankaya KI, Demirok A, Yilmaz OF (2013). Accuracy and precision of cap thickness in small incision lenticule extraction. Clin Ophthalmol.

[29] Yu ZQ, Xu Y, Yao PJ, Qin B, Zhou XT, Chu RY (2010). Analysis of flap thickness by anterior segment optical coherence tomography in different flap preparation styles of excimer laser surgery. Zhonghua Yan Ke Za Zhi.

[30] Yao P, Xu Y, Zhou X (2011). Comparison of the predictability, uniformity and stability of a laser in situ keratomileusis corneal flap created with a VisuMax femtosecond laser or a Moria microkeratome. J Int Med Res.

[31] Ahn H, Kim JK, Kim CK, Han GH, Seo KY, Kim EK, Kim TI (2011). Comparison of laser in situ keratomileusis flaps created by 3 femtosecond lasers and a microkeratome. J Cataract Refract Surg.

[32] Issa A, Al Hassany U (2011). Femtosecond laser flap parameters and visual outcomes in laser in situ keratomileusis. J Cataract Refract Surg.

[33] Reinstein DZ, Archer TJ, Gobbe M (2013). Accuracy and reproducibility of Cap thickness in small incision lenticule extraction. J Refract Surg.

[34] Zhao J, Yao P, Li M, Chen Z, Shen Y, Zhao Z, Zhou Z, Zhou X (2013). The morphology of corneal cap and its relation to refractive outcomes in femtosecond laser small incision lenticule extraction (SMILE) with anterior segment optical coherence tomography observation. PLoS One.

[35] Vestergaard AH, Grauslund J, Ivarsen AR, Hjortdal JO (2014). Central corneal sublayer pachymetry and biomechanical properties after refractive femtosecond lenticule extraction. J Refract Surg.

[36] Tay E, Li X, Chan C, Tan DT, Mehta JS (2012). Refractive lenticule extraction flap and stromal bed morphology assessment with anterior segment optical coherence tomography. J Cataract Refract Surg.

[37] Reinstein DZ, Archer TJ, Gobbe M, Silverman RH, Coleman DJ (2010). Repeatability of layered corneal pachymetry with the Artemis very high-frequency digital ultrasound Arc-scanner. J Refract Surg.

[38] Hall RC, Mohamed FK, Htoon HM, Tan DT, Mehta JS (2011). Laser in situ keratomileusis flap measurements: comparison between observers and between spectral-domain and time-domain anterior segment optical coherence tomography. J Cataract Refract Surg.

[39] Rosas Salaroli CH, Li Y, Zhang X, Tang M, Branco Ramos JL, Allemann N, Huang D (2011). Repeatability of laser in situ keratomileusis flap thickness measurement by Fourier-domain optical coherence tomography. J Cataract Refract Surg.

[40] Carl Zeiss M (2006). Visante OCT User’s manual. Book Visante OCT User’s Manual.

[41] Li Y, Netto MV, Shekhar R, Krueger RR, Huang D (2007). A longitudinal study of LASIK flap and stromal thickness with high-speed optical coherence tomography. Ophthalmology.

[42] von Jagow B, Kohnen T (2009). Corneal architecture of femtosecond laser and microkeratome flaps imaged by anterior segment optical coherence tomography. J Cataract Refract Surg.

[43] Reinstein DZ, Archer TJ, Gobbe M (2014). Lenticule thickness readout for small incision lenticule extraction compared to Artemis three-dimensional very high-frequency digital ultrasound stromal measurements. J Refract Surg.

[44] Reinstein DZ, Archer TJ, Gobbe M (2011). LASIK flap thickness profile and reproducibility of the standard vs zero compression Hansatome microkeratomes: three-dimensional display with Artemis VHF digital ultrasound. J Refract Surg.

[45] Reinstein DZ, Archer TJ, Gobbe M, Silverman R, Coleman DJ (2009). Stromal thickness in the normal cornea: three-dimensional display with Artemis very high-frequency digital ultrasound. J Refract Surg.

[46] Reinstein DZ, Silverman RH, Raevsky T, Simoni GJ, Lloyd HO, Najafi DJ, Rondeau MJ, Coleman DJ (2000). Arc-scanning very high-frequency digital ultrasound for 3D pachymetric mapping of the corneal epithelium and stroma in laser in situ keratomileusis. J Refract Surg.

[47] Roberts C (2000). The cornea is not a piece of plastic. J Refract Surg.

[48] He J, Bazan NG, Bazan HE (2010). Mapping the entire human corneal nerve architecture. Exp Eye Res.

[49] Tuisku IS, Lindbohm N, Wilson SE, Tervo TM (2007). Dry eye and corneal sensitivity after high myopic LASIK. J Refract Surg.

[50] Wilson SE (2001). Laser in situ keratomileusis-induced (presumed) neurotrophic epitheliopathy. Ophthalmology.

[51] Savini G, Barboni P, Zanini M, Tseng SC (2004). Ocular surface changes in laser in situ keratomileusis-induced neurotrophic epitheliopathy. J Refract Surg.

[52] Solomon R, Donnenfeld ED, Perry HD (2004). The effects of LASIK on the ocular surface. Ocul Surf.

[53] Shtein RM (2011). Post-LASIK dry eye. Expert Rev Ophthalmol.

[54] Reinstein DZ, Archer TJ, Gobbe M, Bartoli E (2014). Corneal sensation after small incision lenticule extraction (SMILE). J Refract Surg.

[55] Wei S, Wang Y (2013). Comparison of corneal sensitivity between FS-LASIK and femtosecond lenticule extraction (ReLEx flex) or small-incision lenticule extraction (ReLEx smile) for myopic eyes. Graefes Arch Clin Exp Ophthalmol.

[56] Wei SS, Wang Y, Geng WL, Jin Y, Zuo T, Wang L, Wu D (2013). Early outcomes of corneal sensitivity changes after small incision lenticule extraction and femtosecond lenticule extraction. Zhonghua Yan Ke Za Zhi.

[57] Vestergaard AH, Gronbech KT, Grauslund J, Ivarsen AR, Hjortdal JO (2013). Subbasal nerve morphology, corneal sensation, and tear film evaluation after refractive femtosecond laser lenticule extraction. Graefes Arch Clin Exp Ophthalmol.

[58] Demirok A, Ozgurhan EB, Agca A, Kara N, Bozkurt E, Cankaya KI, Yilmaz OF (2013). Corneal sensation after corneal refractive surgery with small incision lenticule extraction. Optom Vis Sci.

[59] Li M, Zhao J, Shen Y, Li T, He L, Xu H, Yu Y, Zhou X (2013). Comparison of Dry Eye and corneal sensitivity between small incision lenticule extraction and femtosecond LASIK for myopia. PLoS One.

[60] Li M, Zhou Z, Shen Y, Knorz MC, Gong L, Zhou X (2014). Comparison of corneal sensation between small incision lenticule extraction (SMILE) and femtosecond laser-assisted LASIK for myopia. J Refract Surg.

[61] Li M, Niu L, Qin B, Zhou Z, Ni K, Le Q, Xiang J, Wei A, Ma W, Zhou X (2013). Confocal comparison of corneal reinnervation after small incision lenticule extraction (SMILE) and femtosecond laser in situ keratomileusis (FS-LASIK). PLoS One.

[62] Mohamed-Noriega K, Riau AK, Lwin NC, Chaurasia SS, Tan DT, Mehta JS (2014). Early corneal nerve damage and recovery following small incision lenticule extraction (SMILE) and laser in situ keratomileusis (LASIK). Invest Ophthalmol Vis Sci.

[63] Xu Y, Yang Y (2014). Dry eye after small incision lenticule extraction and LASIK for myopia. J Refract Surg.

[64] Knox Cartwright NE, Tyrer JR, Jaycock PD, Marshall J (2012). Effects of variation in depth and side Cut angulations in LASIK and thin-flap LASIK using a femtosecond laser: a biomechanical study. J Refract Surg.

[65] Medeiros FW, Sinha-Roy A, Alves MR, Dupps WJ (2011). Biomechanical corneal changes induced by different flap thickness created by femtosecond laser. Clinics (Sao Paulo).

[66] Randleman JB, Dawson DG, Grossniklaus HE, McCarey BE, Edelhauser HF (2008). Depth-dependent cohesive tensile strength in human donor corneas: implications for refractive surgery. J Refract Surg.

[67] Kohlhaas M, Spoerl E, Schilde T, Unger G, Wittig C, Pillunat LE (2006). Biomechanical evidence of the distribution of cross-links in corneas treated with riboflavin and ultraviolet A light. J Cataract Refract Surg.

[68] Scarcelli G, Pineda R, Yun SH (2012). Brillouin optical microscopy for corneal biomechanics. Invest Ophthalmol Vis Sci.

[69] Petsche SJ, Chernyak D, Martiz J, Levenston ME, Pinsky PM (2012). Depth-dependent transverse shear properties of the human corneal stroma. Invest Ophthalmol Vis Sci.

[70] Winkler M, Shoa G, Xie Y, Petsche SJ, Pinsky PM, Juhasz T, Brown DJ, Jester JV (2013). Three-dimensional distribution of transverse collagen fibers in the anterior human corneal stroma. Invest Ophthalmol Vis Sci.

[71] Reinstein DZ, Archer TJ, Randleman JB (2013). Mathematical model to compare the relative tensile strength of the cornea after PRK, LASIK, and small incision lenticule extraction. J Refract Surg.

[72] Agca A, Ozgurhan EB, Demirok A, Bozkurt E, Celik U, Ozkaya A, Cankaya I, Yilmaz OF (2014). Comparison of corneal hysteresis and corneal resistance factor after small incision lenticule extraction and femtosecond laser-assisted LASIK: a prospective fellow eye study. Cont Lens Anterior Eye.

[73] Kamiya K, Shimizu K, Igarashi A, Kobashi H, Sato N, Ishii R (2014). Intraindividual comparison of changes in corneal biomechanical parameters after femtosecond lenticule extraction and small-incision lenticule extraction. J Cataract Refract Surg.

[74] Wu D, Wang Y, Zhang L, Wei S, Tang X (2014). Corneal biomechanical effects: Small-incision lenticule extraction versus femtosecond laser-assisted laser in situ keratomileusis. J Cataract Refract Surg.

[75] Reinstein DZ, Gobbe M, Archer TJ (2011). Ocular biomechanics: measurement parameters and terminology. J Refract Surg.

[76] Goldich Y, Barkana Y, Morad Y, Hartstein M, Avni I, Zadok D (2009). Can we measure corneal biomechanical changes after collagen cross-linking in eyes with keratoconus?–a pilot study. Cornea.

[77] Touboul D, Roberts C, Kerautret J, Garra C, Maurice-Tison S, Saubusse E, Colin J (2008). Correlations between corneal hysteresis, intraocular pressure, and corneal central pachymetry. J Cataract Refract Surg.

[78] Riau AK, Ang HP, Lwin NC, Chaurasia SS, Tan DT, Mehta JS (2013). Comparison of four different VisuMax circle patterns for flap creation after small incision lenticule extraction. J Refract Surg.

[79] Mohamed-Noriega K, Toh KP, Poh R, Balehosur D, Riau A, Htoon HM, Peh GS, Chaurasia SS, Tan DT, Mehta JS (2011). Cornea lenticule viability and structural integrity after refractive lenticule extraction (ReLEx) and cryopreservation. Mol Vis.

[80] Angunawela RI, Riau AK, Chaurasia SS, Tan DT, Mehta JS (2012). Refractive lenticule re-implantation after myopic ReLEx: a feasibility study of stromal restoration after refractive surgery in a rabbit model. Invest Ophthalmol Vis Sci.

[81] Lim CH, Riau AK, Lwin NC, Chaurasia SS, Tan DT, Mehta JS (2013). LASIK following small incision lenticule extraction (SMILE) lenticule re-implantation: a feasibility study of a novel method for treatment of presbyopia. PLoS One.

[82] Barraquer JI (1980). Queratomileusis y queratofakia.

[83] Barraquer JI (1972). Keratophakia. Trans Ophthalmol Soc U K.

[84] Kaufman HE, McDonald MB (1985). Refractive surgery for aphakia and myopia. Trans Ophthalmol Soc U K.

[85] Liu H, Zhu W, Jiang AC, Sprecher AJ, Zhou X (2012). Femtosecond laser lenticule transplantation in rabbit cornea: experimental study. J Refract Surg.

[86] Pradhan KR, Reinstein DZ, Carp GI, Archer TJ, Gobbe M, Gurung R (2013). Femtosecond laser-assisted keyhole endokeratophakia: correction of hyperopia by implantation of an allogeneic lenticule obtained by SMILE from a myopic donor. J Refract Surg.

[87] Yao P, Zhao J, Li M, Shen Y, Dong Z, Zhou X (2013). Microdistortions in Bowman’s layer following femtosecond laser small incision lenticule extraction observed by Fourier-domain OCT. J Refract Surg.

[88] Dong Z, Zhou X, Wu J, Zhang Z, Li T, Zhou Z, Zhang S, Li G (2014). Small incision lenticule extraction (SMILE) and femtosecond laser LASIK: comparison of corneal wound healing and inflammation. Br J Ophthalmol.

[89] Agca A, Ozgurhan EB, Yildirim Y, Cankaya KI, Guleryuz NB, Alkin Z, Ozkaya A, Demirok A, Yilmaz OF (2014). Corneal backscatter analysis by in vivo confocal microscopy: fellow eye comparison of small incision lenticule extraction and femtosecond laser-assisted LASIK. J Ophthal.

